# On the Action of Light in Small-Pox

**Published:** 1871

**Authors:** J. H. Waters


*On the Action of Light in Small-Pox.* By J. H. WATERS, M.B.

NUMBER II.

The action of light on vegetable nature is more easily studied than that on animal, its influence being, for a time, at least, of more vital importance to the one than to the other. An excellent example of its effect is seen in Priestley's "green matter," which forms on water and damp places when they are exposed to the sunlight, and which consists of cells of the Confervæ in various stages of development ; for although a certain amount of heat is necessary to their formation, it is to light their existence is due, both in the first place and during their process of life. No degree of heat without light will produce these cells, however much the air may be charged with the germs from which they are formed. On water of any degree of temperature, placed in the dark, there is no formation whatever to be found; but if light is freely admitted, variations in the heat of the water is of little consequence, and under the same degree of heat the formation depends on the intensity of the light. Plant-seeds allowed to grow in the dark are only developed to a certain extent; their leaves, etc., are found to be of a yellowish white, and their solid contents do not increase in weight, though their bulk may enlarge by the absorption of water; but if these plants are examined with care when exposed to the sunlight, they are found to be constantly decomposing the carbonic acid of the atmosphere, appropriating the carbon, and setting free the oxygen. For them to carry out this function they must be subjected to the light-giving rays of the solar spectrum, and in proportion to the illuminating power of the ray, so do they perform this action. It is also found that the stomata (orifices in the cuticle of the leaves of the higher plants, for the admission of air, etc.,) have two or more cells that guard their entrance, and perform the action of closing or of opening them; the former, when the plant is in the dark, and the latter, when light pervades the medium in which they are placed. These orifices perform for the vegetables in which they are found an analogous function to the sudoriparous glands of the animal. Thus, the green surfaces of plants grow towards the light, on account of the operations they have to perform under its influence; the roots avoid the light, as they throw out the carbonic acid by respiration; for if light is thrown on germinating seeds from below, the roots grow upwards, and the stems, etc., downwards.

On animals, the action of light is little known. It is evident it differs from that on vegetables in their first stage of nutrition, because animals do not perform any such acts of combination; but, by assimilating the vegetables themselves, obtain their nourishment so elaborately prepared for them by these, as it were, intermediate links in the chain or "circle of life." Animal surfaces do not, therefore, become so extended as those of the vegetable; still, there is no doubt that the health and well-being of animals do also, in a great measure, depend upon light. We know their color is derived from its action, for the eyes and hair of the newly born are without any decided color for days after their birth. When an animal has been found embeded in a stone, it is transparent, and so sensitive that it will hide from the light if exposed to it. In the races of men where the sunlight acts upon their skin, we find freckles, a swarthy hue, and different degrees of pigmentary development up to black, according as there is more or less intensity in the light of the country in which they and their ancestors lived. The pigment cells in the skin, to which the color is due, are like true epidermis cells drying up and becoming flattened scales as they near the surface, thus imparting the blackness to it. They are best seen in their integrity when covering the blood-vessels of the choroid coat of the eye; here they present the polygonal form; each cell contains a nuclus, and the dark color is caused by numbers of flattened round granules. Their chemical nature is not known; it is shown to resemble very nearly the cuttle-fish ink or sepia, which derives its color from the cells lining the ink-bag of the animal, and to have more carbon than most of the organic substances (about 58½ parts in 100).

Many researches show that ligh greatly modifies the development of animals, their health, and recovery from disease. Dr. Edwards has proved the first; for if tadpoles are properly fed, and have plenty of water, but are kept in the dark, he has remarked they never undergo the change into air-breathing animals, but remain fish—large tadpoles. The appearance of animalcules in infusions of decaying organic matter is greatly retarded by being placed in the dark. If those animals which inhabit dark places, as the cockroach, are reared entirely secluded from light, they grow up almost colorless, like plants under similar circumstances.

Nations who inhabit hot climates, and wear little clothing, are remarkably free from deformity ; on the other hand, those people who live in mines, in cellars, or in dark, narrow streets, have mostly mis-shapen and weakly children. This may be partly due to bad air, but greatly to insufficient light. Patients recover better from most diseases when their apartments are properly lighted. Even in health the effect of the dark days is felt on the spirits of every one, more or less.

The circulating fluid is the means by which all parts of the body are nourished. In man, the blood's component parts are fibrine, albumen, corpuscles, saline matters, and ill-defined animal principles. When these sources are duly supplied, and when all the various organs and tissues abstract from it the special materials they need, a general balance is maintained in the system, and, as Mr. Paget has so well said, "each single part of the body, in respect of its nutrition, stands to the whole body in the relation of an excreted substance." But allow any of the rough materials for forming the blood to be too large or too small in quantity, or let an organ act imperfectly, and every part of the body becomes more or less unfavorably affected. In disease of the skin, its sensibility is greatly altered, according as the peripheral termination of the sensory nerves in it are supplied with blood; anything that retards this supply diminishes their sensibility, and if the flow be completely stopped, entire loss of feeling results, as in severe and prolonged cold. If there is a determination of blood to the surface, it becomes highly sensitive, and it is more easily acted upon by external stimulants, as cold, heat, light, etc.; the nerves being supernaturally supplied with nourishment, they convey to the brain perverted impressions. Thus the temperature of the part does not really increase beyond that of the left ventricle of the heart; but the greater impressibility of the nerves leads the patient to imagine it does; there is greater appreciation of the heat, and even magnifying of the degree. Pain also is due to their excited state, and to pressure upon the terminal branches by the dilated blood-vessels. In disease nature frees herself from the morbific poison by the action of the glands, and of these, perhaps, the most used are the sudoriparous and the sebaceous. It is by these the poison is eliminated in small-pox; but by their excessive action inflammation is set up, the nerves become hypersensitive, they magnify the effect of external stimulants, and by reflex action the inflammation of the part is increased, when thus exposed to what in ordinary circumstances would be but healthy and desirable stimulation; the reflex inflammation preventing the natural elimination of the disease, and the two causes increasing its severe symptoms. The tissues thus injured approximate to the condition of dead matter; and, as Mr. Lister has shown, pus is thus formed, which, being re-absorbed, causes secondary fever —that is, true surgical fever, as Mr. J. E. Erichsen says, "occurring only as a consequence of, and secondary to, local disease or injury." The stimulating action of light has a determined effect in this disease, and for this reason I have followed John of Gadsen's idea, excluding it from the patient in the eruptive stage. In practice I find the most beneficial results to follow, rendering what has hitherto been so dangerous and loathsome, comparatively mild and inoffensive. It is wonderful to see a patient with severe confluent small-pox on face and body, sitting up, with no fever, no head symptoms, no pain, enjoying her food, no itching, tingling, or bad smell, or in fact, aught but a stiff, strange feel of the skin, which is described as "rather unpleasant," followed by no secondary fever, and no other complications; and yet this is the history of a case I attended a few weeks back, and is the description of all, even the most severe cases.

What the chemical action of light may be, is not yet known. There is little doubt that both animals and vegetables depend upon it for their well-being in a far greater measure than is supposed.—*Lancet.*

March 30th, 1871.

